# Epidemiological characterization of incident cases of *Rickettsia* infection in rural areas of Urabá region, Colombia

**DOI:** 10.1371/journal.pntd.0006911

**Published:** 2018-10-31

**Authors:** Juan Carlos Quintero Vélez, Daniel Camilo Aguirre-Acevedo, Juan David Rodas, Margarita Arboleda, Adriana Troyo, Francisco Vega Aguilar, Lisardo Osorio Quintero, Carlos Rojas Arbeláez

**Affiliations:** 1 Grupo de Investigación Ciencias Veterinarias Centauro, Facultad de Ciencias Agrarias, Universidad de Antioquia, Medellín, Colombia; 2 Instituto de Investigaciones Médicas, Facultad de Medicina, Universidad de Antioquia, Medellín, Colombia; 3 Instituto Colombiano de Medicina Tropical- CES, Apartadó, Antioquia, Colombia; 4 Centro de Investigación en Enfermedades Tropicales, Facultad de Microbiología, Universidad de Costa Rica, San José, Costa Rica; 5 Grupo Salud y Ambiente, Facultad Nacional de Salud Pública, Universidad de Antioquia, Medellín, Colombia; 6 Grupo de Epidemiología, Facultad Nacional de Salud Pública, Universidad de Antioquia, Medellín, Colombia; Northern Arizona University, UNITED STATES

## Abstract

**Introduction:**

Most of the studies related to rickettsial infection in Colombia are cross-sectional because of the challenge in conducting prospective studies on infectious disease that may have a difficult diagnosis. Although cross-sectional studies are essential to detect people exposed to rickettsiae, they are not suited to demonstrate the recent circulation of this pathogen in areas at risk of transmission.

**Objective:**

To characterize the epidemiology of incident cases of Spotted fever group (SFG) rickettsial infection in humans and equines from rural areas of Urabá region in Colombia where outbreaks of rickettsiae previously occurred.

**Materials and methods:**

A prospective study was conducted in the Alto de Mulatos and Las Changas in the Urabá region. Serum samples and socio-ecological information were collected from 597 people enrolled in 2015, and a second sample was collected from 273 people a year later. Indirect immune-fluorescence assays for detection of IgG antibody against rickettsiae were done using slides with *Rickettsia rickettsii* antigens. A titer ≥128 was considered positive. Incident cases were defined as (i) serological conversion of IgG titers from seronegative to seropositive or (ii) at least a four-fold increase in IgG end point titers in the second sample.

**Results:**

The cumulative incidence of rickettsial infection was 6.23% (95%CI 3.67–9.78) in humans and 32.31% (21/65) of incident cases in equines. Incident cases were mostly females (82.35%), the median age of cases was 41.02 years (IQR 18.62–54.1), and 29.41% reported tick bites during the study period. Results from multivariate analysis showed that removal of ticks after working outdoors is a protective factor for rickettsial infection (RR 0.26, 95%CI 0.08–0.84) and that a higher incidence of infection occurred in people who reported fever in the last year (RR 4.26, 95%CI 1.15–9.31).

**Conclusions:**

These results showed recent circulation of SFG rickettsiae in areas where previous lethal outbreaks have been reported, supporting the implementation of preventive measures to halt rickettsial transmission in the studied communities.

## Author summary

On the northwestern coast of Colombia three lethal outbreaks of rickettsioses caused by the species *Rickettsia rickettsii* have been reported. The lack of an active epidemiological surveillance system in these areas makes difficult the detection and the timely treatment of the disease prompting a worse prognosis. In this study, new cases of rickettsial infection were detected in people and horses living in areas where outbreaks of the disease occurred in the previous decade. In addition, the results showed that nonspecific febrile syndromes are associated to rickettsial infection and protective measures such as tick removal from the body decrease the risk of infection. The design, evaluation and implementation of a surveillance program of emerging and re-emerging infectious diseases, such as rickettsioses, should be a priority in the country, especially in areas where such infections have proved to be lethal.

## Introduction

Epidemiological surveillance of febrile syndromes in the Urabá region of Colombia is currently focused on malaria, leptospirosis, and dengue, and recently, Zika and chikungunya due to epidemics in the area during 2015 and 2016. Additional febrile syndromes are under diagnosed because of their unspecific signs and symptoms, as well as the lack of a rapid diagnostic test in the acute phase of the disease; consequently, they are classified as “unspecific febrile syndromes” in the regional surveillance reports.

Ricketssiosis is one of the most challenging diseases in marginalized regions of Colombia because it is not considered in the differential diagnosis of febrile syndromes, neither in low nor high complexity medical centers. The neglected status of the disease causes delays in effective treatments and consequently produces high lethality among infected patients [1–3]. The problem can be averted with the implementation of surveillance programs to detect rickettsial diseases in Colombia, and the prescription of doxycycline as an effective treatment recommended by the CDC [4].

Furthermore, the diagnosis of rickettsiosis is often difficult because (i) to confirm a rickettsial case two samples from the acute and convalescent phases of the disease (15 to 20 days after the beginning of symptoms) must be obtained, and the latter is seldom collected because patients are lost to the follow-up [5]; (ii) suspected cases are usually confirmed using molecular techniques which are not available in most medical centers in the country; (iii) bacterial detection depends on the stage of the disease, and when the sample is not collected during the period of rickettsemia, a false negative result can occur [4]; and (iv) when patients died from an unspecific febrile syndrome, samples of affected tissues are rarely collected for post-mortem diagnosis in specialized laboratories [4]. The challenging diagnosis of rickettsiosis limits the knowledge about the disease and produces gaps in disease burden, incidence of the infection/disease, vectors implicated, amplifying hosts, and places and time of high risk for transmission.

Most of the studies on rickettsioses in Colombia are cross-sectional and descriptive [6–8]. These investigations have addressed ecological and sociodemographic processes related to the seroprevalence of infection; however, they are unable to determine space-temporal relationships between infection and disease. Therefore, the aim of this study was to characterize the epidemiology of incident cases of SFG rickettsial infection in humans and equines from Urabá region in Colombia, where lethal outbreaks of diseases had occurred previously [1,2]. The tested hypothesis was that incident cases of rickettsial infections occur in humans and equines in the rural areas of Urabá, and these cases are related to socio-ecological characteristics.

## Materials and methods

### Location

The study was conducted in Alto de Mulatos in the municipality of Turbo (8°08'12.5"N 76°33'01.7"W), and Las Changas in the municipality of Necoclí (8°32'52.5"N 76°34'23.7"W). The urban center and hamlets are found in both sites, although some hamlets are usually difficult to access because of the long distance to the urban center. The economy is based on rice, corn, yam, yucca, and cacao crops, and not specialized livestock. Recently, lands previously used for crops and livestock are being replaced with teca trees (*Tectona grandis*) changing the ecosystem of this area [9].

### Study design

A prospective study was designed including 597 people, 342 from Las Changas and 255 from Alto de Mulatos. In addition, 136 equines, 71 from Las Changas and 64 from Alto de Mulatos, were included (T0) [10]. At baseline (T0; year 2015), blood samples from people and equines were taken, and information from an epidemiological survey was collected [10]. Accordingly, in the previous study (T0), nine hamlets were selected for convenience based on ease of access to the hamlets, shorter distance to the urban center, public safety, number of households and ecological conditions favorable for rickettsiae transmission, such as the presence of domestic animals, opossums, wild or synanthropic rodents, and previous reports of humans bitten by ticks. A finite probabilistic complex sample was designed, in which the sample units were households within the nine hamlets and analysis units were people inhabiting the households. The sample size calculations indicated that 208 households inhabited by 865 persons would suffice to detect associations with a 95% confidence level, considering 5% error and 41% expected prevalence of infection in humans [8]. Sample size was calculated in Epidat 4.0 [11]. Households within each hamlet were selected using probability proportional to size sampling. In addition, non-probability sampling of domestic animals was done in the nine hamlets to study the role of canines and equines as sentinels of infection.

A year later (T12; year 2016), a second blood sample from humans and equines was collected by house-to-house visits. Also, an epidemiological survey was conducted to evaluate changes in exposures in the last year such as occupation, tick exposure, fever episodes, and the presence of domestic animals in households.

### Inclusion and exclusion criteria

Participants from any age and both sexes who agreed to participate and signed the informed consent, and who resided in the following sites were included: the urban center of Las Changas and its hamlets: La Union, El Salado and El Cativo; and the urban center of Alto de Mulatos and its hamlets: Juan Benitez, Quebrada del Medio, Caracolí and La Trampa. People were excluded if they were considering moving out from the study area within the following year, or people who were suspected to be affiliated to illegal army groups.

### Outcome, exposures and covariates

The main outcome was the incident cases of SFG rickettsial infection in people. The outcome was defined as (i) serological conversion of IgG titers from seronegative (T0) to seropositive (with an end point titer ≥128) (T1) or (ii) at least four-fold increase in IgG end point titers in the second blood sample (T1) compared to the first blood sample (T0). The same definitions were applied to incident cases in equines.

The main exposures analyzed in the study were occupation and tick infestation in the previous year. Occupation was categorized as outdoors and indoors; farmers, ranchers, and similar jobs were considered outdoors occupations, and the remaining were considered indoors occupations. In addition, information about changing covariates in the follow-up year were included, such as fever episodes and presence of domestic animals (canines and equines) in intra- and peri-domiciliary areas. Age in years and sex were considered as confounders of main exposures according to results from the cross-sectional analysis done at the beginning of the study [10].

Additional covariates of interest were materials used for building the households (roofs, floors, and walls), characteristics of peri-domiciliary areas (rice, corn, tomato, yam, yucca, and cacao crops) and the presence of domestic animals, in addition to canines and equines (swine, poultry, turkey, bovines). Finally, family protection practices against tick infestation were recorded, such as use of long sleeved and white shirts for working outdoors, use of repellents against insects, and appropriate handling of companion animals (bathing and antiparasitic treatment).

### Serological testing

IgG antibody titers against SFG rickettsiae were evaluated using indirect immune-fluorescence assay (IFA) with slides containing *Rickettsia rickettsii* antigens. According recommendations to detecting infection in rickettsial endemic areas [12], a serum dilution of 1/128 was considered the cut-off point. All positive samples from humans and equines collected during T0 were tittered until the end point of positivity.

In addition, to detect the species probably infecting humans and equines, seropositive samples were analyzed using slides with antigens of *R*. *parkeri*, *R*. *amblyommatis*, *R*. *belli*, *R*. *rhipicephali* and *R*. *felis*. The species probably infecting humans/equines was defined according to a four-fold increase in IgG antibody end point titers compared to the titers detected in the remaining species evaluated [13,14].

### Statistical analysis

The cumulative incidence was estimated considering the number of incident cases in the numerator and the number of people evaluated in both time T0 and T12 as denominator. Incident cases in humans and equines were characterized using relative and absolute frequencies for qualitative variables, and median and interquartile range for quantitative variables.

Risk or protective factors associated with rickettsial infection were estimated using a mixed effects logistic regression model [15]. Household level was considered the random effect and a variance component correlation matrix was used. The multivariate model was built including variables with p<0.25 in the bivariate analysis [16], and selection of variables were done using the stepwise method. Sex and age (in years) were fixed in the model according to the results from the cross-sectional study [10], because both variables were confounders of the association between seropositivity and the main outcome (working outdoors). All analyses were performed in SAS 9.04.01 [17].

Odds ratios were transformed in relative risks using the following function [18]:

$$RR=(1+e^{\left(-b_{0}\right)})/(1+e^{\left(-b_{0}-b_{1}\right)})$$

where:

e = is the base of Napierian logarithms

b_0_ = regression model intercept

b_1_ = regression model parameter

### Georeferencing of incident cases

Human and equine incident cases were georeferenced using the package for managing the geographic information systems ArcGis 10.4.1.

### Ethical considerations

The Bioethics Committee of the Facultad Nacional de Salud Pública and the Ethics Committee for animal experimentation of Universidad de Antioquia approved all procedures performed in this study. All adult participants (>18years) signed a informed consent. Children (< 18 years) were enrolled in the study after parents or guardians signed the informed consent on their behalf. The informed consent was signed during the first visit to households for sample collection at T0 and T12. The animal protocol used in this study adhered to the Colombian Law 84 of 1989 regulating the protection of animals against suffering and pain in the Colombian territory.

## Results

### Incidence of rickettsial infection and characterization of incident cases in humans

**Fig 1:**
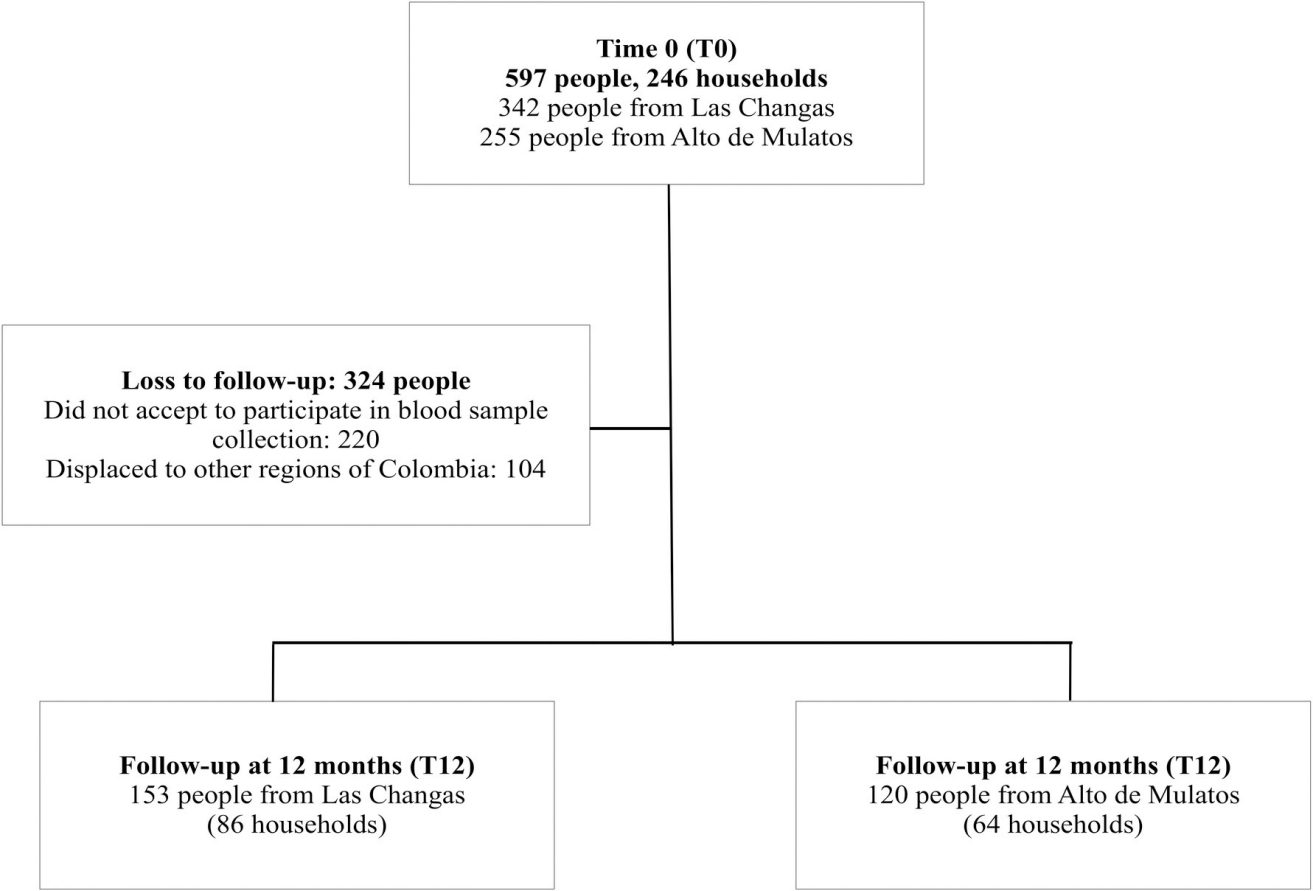
Flowchart of people enrolled in the study at T0 and T12.

From 597 people enrolled in the study at time zero (November 2015-January 2016), 273 were followed-up twelve months later (November 2016-January 2017). The seroprevalence at T0 was 25.62% (153/597) (95% CI 22.11–29.12). People were lost at follow-up mainly because their displacement to other regions of Colombia. Of the people followed-up, 153 were from Las Changas and 120 from Alto de Mulatos. People included in the study inhabited 150 households, 86 located in Las Changas and 64 located in Alto de Mulatos (Fig 1). The cumulative incidence of rickettsial infection was estimated in 6.23% (17/273) (95%CI 3.67–9.78).

Baseline characteristics of participant followed-up and lost to follow-up at T12 are presented in Table 1.

**Table 1:** Sociodemographic characterization of people enrolled in the study at T0 and followed-up at T12 in Las Changas and Alto de Mulatos, Colombia.

Baseline characteristics	Followed-up at T12 Alto de Mulatos (n = 120)	Lost to follow-up at T12 Alto de Mulatos (n = 135)	Followed-up at T12 Las Changas (n = 153)	Lost to follow-up at T12 Las Changas (n = 189)
Age, in years (median, IQR)	33,14 (16,3–47,7)	23,8 (12,9–39,9)	36,0 (20,4–53,8)	28,3 (13,9–44,8)
Male sex	30%	54,07%	31,37%	40,74%
Working outdoors	15%	24,44%	29,41%	30,69%
Household location (rural)	45%	42,22%	61,4%	52,38%
Tick infestation	38,66%	91,85%	10,46%	87,83%
Fever episodes	9,17%	51,11%	1,96%	61,38%
Removal of ticks	66,67%	74,07%	50,33%	35,45%
Deforestation for agriculture use	66,67%	86,67%	59,48%	47,62%
Presence of opossum in peridomiciliary area	53,33%	32,84%	57,24%	50,54%

Seven new cases of rickettsial infection were detected in Las Changas, and 10 new cases of infection in Alto de Mulatos. Sociodemographical caracteristics of incident cases are presented in Table 2.

**Table 2:** Sociodemographic characteristics of rickettsial incident cases in Las Changas and Alto de Mulatos, Colombia.

Variable	Alto de mulatos (n = 7)	Las Changas (n = 10)	Total (n = 17)
Age (years)	31,83 (17,2–54,1)	48,28 (27,8–54,4)	41,02 (18,62–54,1)
Female sex	100%	70%	82,35%
Working outdoors	14,29%	50%	35,29%
Removal of ticks	42,86%	20%	29,41%
Location of household (rural)	71,43%	30%	47,06%
Fever episodes	28,57%	10%	17,65%
Tick infestation	28,57%	30%	29,41%

### Characterization of rickettsial incident cases in equines

**Fig 2:**
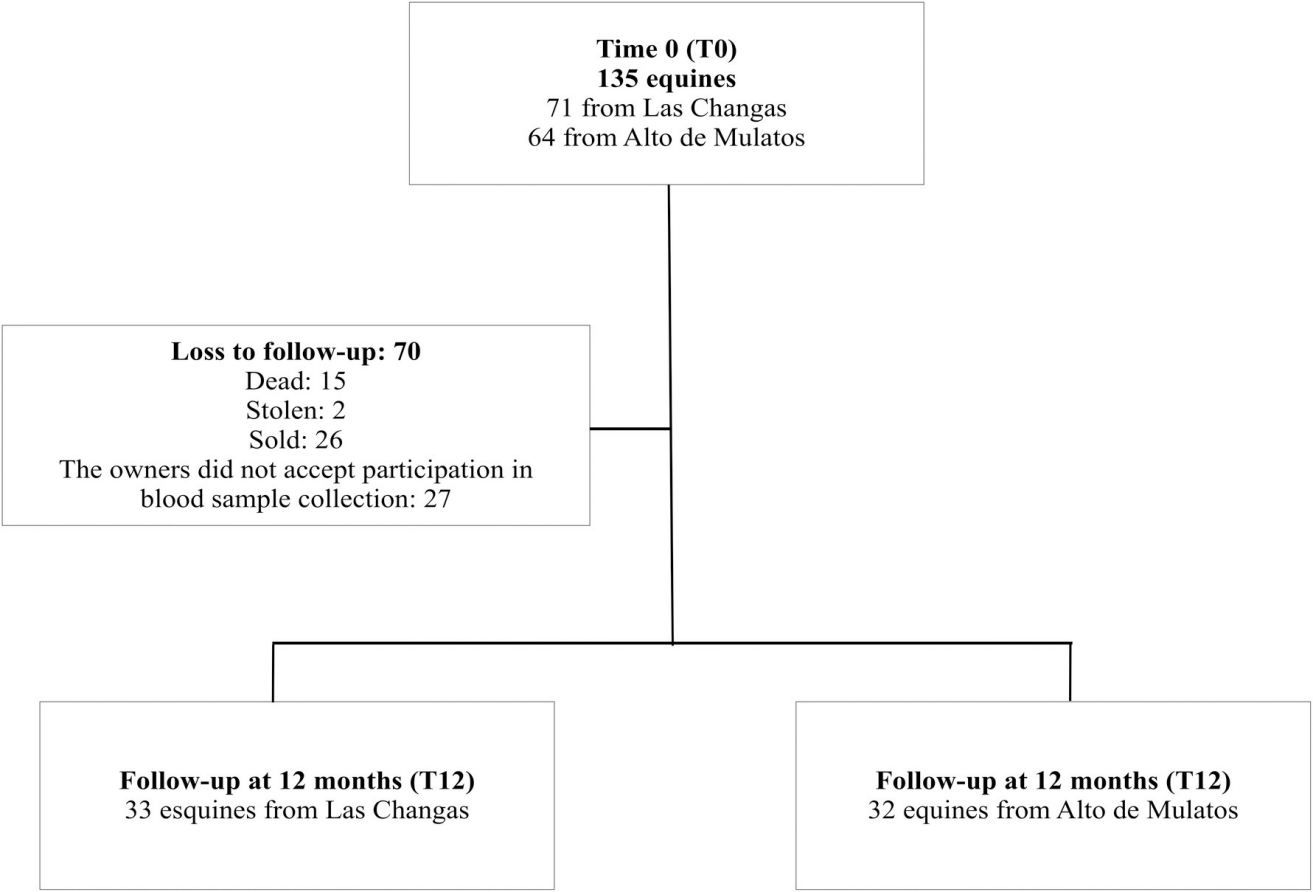
Flowchart of equines included in the study at T0 and T12.

One hundred and thirty-five equines were included in T0 (January 2016), 71 from Las Changas and 64 from Alto de Mulatos. At T12 (January 2017), a blood sample was collected in 65 out 135 equines included in T0, 33 equines were from Las Changas and 32 from Alto de Mulatos. Most equines were lost at follow-up because were dead, stolen, sold or the owners did not accept to participate in blood sample collection (Fig 2).

The proportion of incident cases of rickettsial infection in equines was 33.85% (22/65). Among incident equines, 50% (11/22) were from Las Changas, and the remaining animals from Alto de Mulatos. General characteristics of equines incident for rickettsial infection are presented in Table 3.

**Table 3:** Characteristics of incident cases of rickettsial infection in equines.

Code	Species	Age (in years)	Sex	Site
18	*E*. *caballus*	7	Female	Las Changas (UC)
47	*E*. *asinus*	6	Male	La Unión
50	*E*. *asinus*	9	Male	La Unión
52	*E*. *caballus*	9	Female	La Unión
62	*E*. *asinus*	6	Female	La Unión
66	*E*. *asinus*	8	Female	La Unión
70	*E*. *asinus*	15	Female	La Unión
72	*E*. *asinus*	13	Female	La Unión
104	*E*. *asinus*	5	Female	Las Changas (UC)
129	*E*. *asinus*	3	Male	Cativo
131	*E*. *asinus*	6	Male	Cativo
146	*E*. *caballus*	9	Female	Alto de Mulatos (UC)
162	*E*. *caballus*	-	Male	Alto de Mulatos (UC)
163	*E*. *caballus*	-	Male	Alto de Mulatos (UC)
170	*E*. *asinus x E*. *caballus*	11	Male	Alto de Mulatos (UC)
214	*E*. *caballus*	9	Male	Caracolí
215	*E*. *asinus x E*. *caballus*	26	Male	Caracolí
220	*E*. *caballus*	-	Male	Caracolí
222	*E*. *asinus x E*. *caballus*	-	Female	Caracolí
229	*E*. *caballus*	2	Female	Caracolí
232	*E*. *asinus x E*. *caballus*	7	Male	Caracolí
235	*E*. *caballus*	8	Male	Caracolí

UC: urban center

### Rickettsial species infecting incident cases

Serological assays using different rickettsial antigens (*R*. *parkeri*, *R*. *rickettsii*, *R*. *amblyommatis*, *R*. *belli*, *R*. *rhipicephali*, *R*. *felis*) were not able to detect the species probably infecting humans. Only three samples showed high antibody end point titers against both *R*. *rickettsii* and *R*. *amblyommatis* (Table 4).

**Table 4:** Antibody titers of incident cases of rickettsial infection in people using different *Rickettsia* antigens.

Code	*R*. *rickettsii*	*R*. *amblyommatis*	*R*.*felis*	*R*.*parkeri*	*R*.*rhipicephali*	*R*.*belli*
146	128	128	256	<256	<256	<256
168	512	256	128	<512	<512	<512
**177**	**2048**	**2048**	**1024**	**<2048**	**<2048**	**<2048**
208	512	128	512	<512	<512	<512
294	128	128	128	<128	<128	294
307	128	512	256	<512	<512	<512
346	512	512	128	<512	<512	512
**464**	**1024**	**1024**	**128**	**<1024**	**<1024**	**<1024**
469	256	128	128	<256	<256	<256
506	512	128	256	<256	<256	<256
521	256	256	256	<256	<256	<256
549	256	128	128	<256	<256	<256
559	512	512	128	<512	<512	<512
**567**	**2048**	**2048**	**256**	**<2048**	**<2048**	**<2048**
568	256	256	128	<256	<256	<256
630	128	256	128	<256	<256	<256
657	256	256	256	<256	<256	256

Two out of three people with high IgG titers against *R*. *rickettsii* and *R*. *amblyommatis* were from hamlet La Salada and the urban center of Las Changas. The remaining person was from hamlet Caracolí of Alto de Mulatos.

Regarding equines, it was possible to detect the infecting species in seven out of 22 incident cases. *R*. *amblyommatis* probably infected four equines from hamlet La Union of Las Changas and one from the urban center of Alto de Mulatos. *R*. *rickettsii* probably infected two equines, one from hamlet La Unión of Las Changas and the other from the urban center of Alto de Mulatos. In addition, one equine from hamlet Caracolí of Alto de Mulatos was probably infected with *R*. *belli* (Table 5). Finally, two equines from hamlets La Unión and Cativo of Las Changas showed high IgG end point titers against both *R*. *amblyommatis* and *R*. *rickettsii*, and thus it was not possible to identify the infecting species (Table 5).

**Table 5:** IgG antibody titers against *Rickettsia* species infecting equines incident cases.

Code	*R*. *rickettsii*	*R*. *amblyommatis*	*R*. *felis*	*R*. *parkeri*	*R*. *rhipicephali*	*R*. *belli*
50 Unión	**4096**	512	256	<1024	<1024	<1024
62 Unión	128	**512**	<128	<128	<128	<128
70 Unión	<128	**512**	<128	<128	<128	<128
72 Unión	128	**512**	<128	<128	<128	<128
146 AM (UC)	**256**	<128	<128	<128	<128	<128
163 AM (UC)	<128	**256**	<128	<128	<128	<128
222 Caracolí	128	128	128	<128	<128	**512**
47 Unión^a^	**2048**	**2048**	128	8192 (-)	8192 (-)	8192 (-)
131 Cativo^a^	**2048**	**4096**	512	<128	<128	128

AM: Alto de Mulatos, UC: urban center

^a^Equines with high IgG antibody titers against both *R*. *rickettsii* and *R*. *amblyommatis* in which was not possible to determine the rickettsial species infecting the equine.

**Fig 3:**
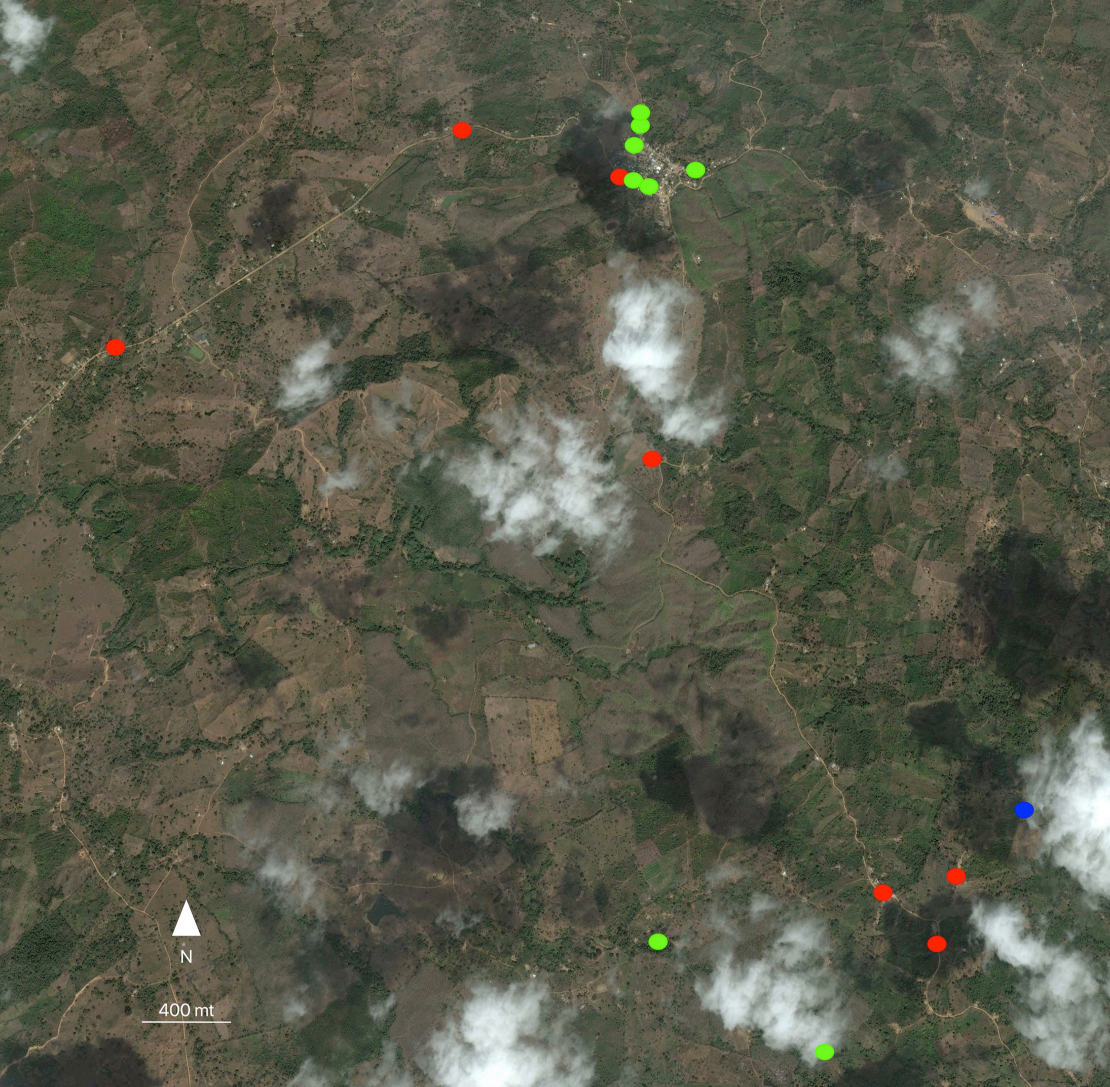
Georeferencing of incident cases of rickettsial infection in people and equines in Las Changas, including equines probably infected with *R*. *rickettsii* and *R*. *amblyommatis*. Incident cases of rickettsial infection in humans (green circles) and equines (red circles), equines probably infected with *R*. *rickettsii* (blue circles) and *R*. *amblyommatis* (pink circles) (ArcGis 10.4.1).

**Fig 4:**
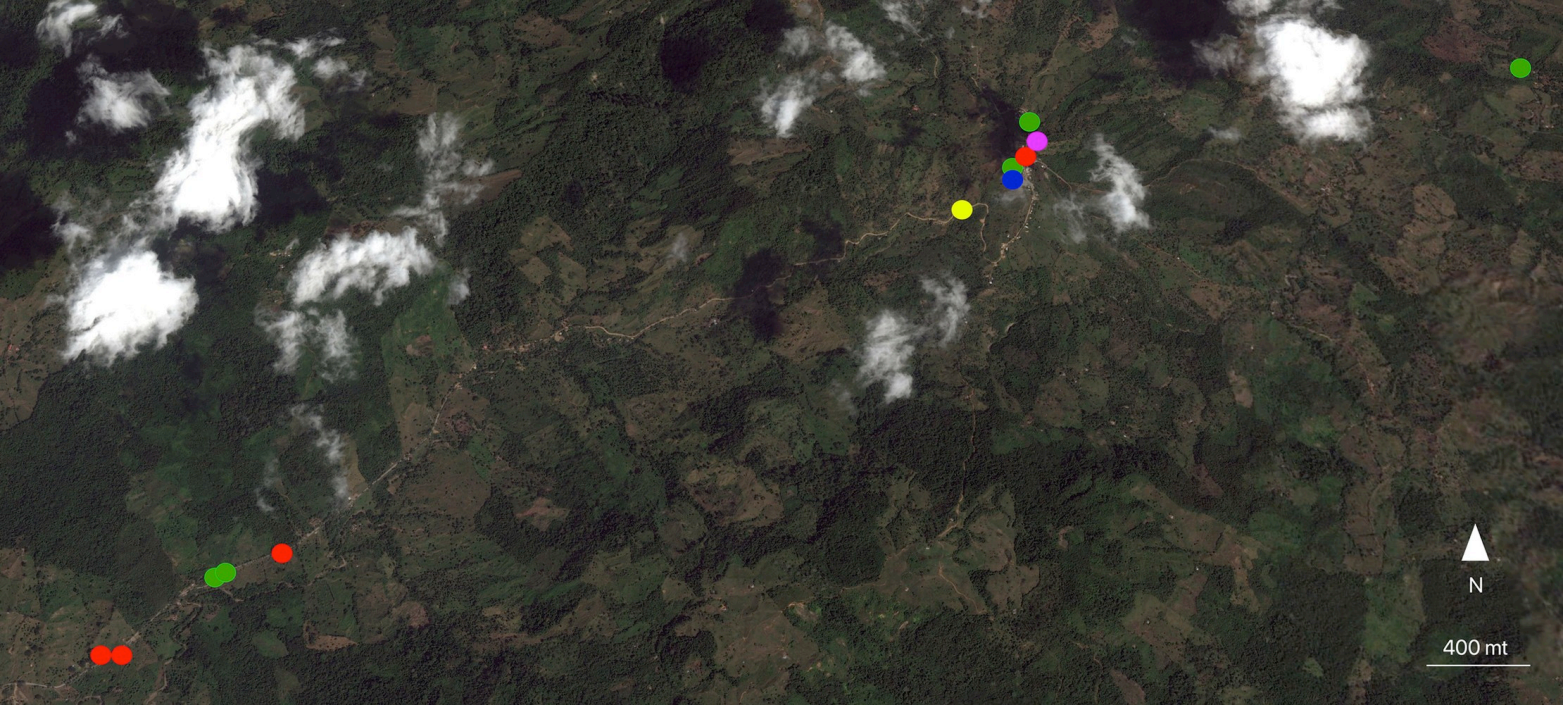
Georeferencing of incident cases of rickettsial infection in people and equines in Alto de Mulatos, including equines probably infected with *R*. *rickettsii* and *R*. *amblyommatis*. Incident cases of rickettsial infection in humans (green circles) and equines (red circles), equines probably infected with *R*. *rickettsii* (blue circles) and *R*. *amblyommatis* (pink circles) (ArcGis 10.4.1).

The geographical distribution of incident cases of rickettsial infection in people and equines, including equines probably infected with *R*. *rickettsii* and *R*. *amblyommatis* are showed in Figs 3 and 4.

### Bivariate and Multivariate analysis of incident cases of rickettsial infection

The following variables were included in the multivariate mixed effects logistic regression model: occupation, fever episodes in the last year, sites at risk (grasses, bushes, forest, among others), removal of ticks, household materials (dirt soil and zinc roofs), and presence of domestic animals (felines, porcines and poultry) (Table 6).

**Table 6:** Descriptive, bivariate and multivariate mixed effects logistic regression analysis of incident cases of rickettsial infection.

Variables	Total n = 273 n(%)	Incident n = 17 n(%)	Non-incident n = 256 n(%)	Crude RR (95%CI)	Adjusted RR (95%CI)
**Individuals**					
Occupation (outdoors)	63 (23,08)	6 (35,39)	57 (22,27)	1,92 (0,64–5,25)	
Female sex	189 (69,23)	14 (82,35)	175 (68,36)	2,09 (0,56–7,39)	
Age, years (median, IQR)	34,68 (18,35–51,88)	41,02 (18,63–54,12)	34,26 (18,32–51,38)	1,01 (0,98–1,03)	
Tick infestation	62 (22,79)	5 (29,41)	57 (22,35)	1,42 (0,44–4,02)	
Fever (last year)	14 (5,13)	3 (17,65)	11 (4,30)	4,37 (1,06–12,63)	4.26 (1.15–9.31)
Sites at risk	171 (63,10)	8 (47,06)	163 (64,17)	0,94 (0,19–1,42)	
**Roof material**					
Zinc	179 (65,57)	14 (82,35)	165 (64,45)	2,45 (0,66–8,13)	
Vegetal	136 (49,82)	9 (52,94)	127 (49,61)	1,13 (0,39–3,15)	
Wood	24 (8,79)	0 (0,0)	24 (9,38)	NE	
Tile	15 (5,49)	0 (0,0)	15 (5,86)	NE	
**Floor material**					
Dirt soil	187 (68,50)	14 (82,35)	173 (67,58)	2,20 (0,58–7,56)	
Cement	111 (40,66)	6 (35,29)	105 (41,02)	0,82 (0,27–2,39)	
Wood	23 (8,42)	1 (5,88)	22 (8,59)	0,71 (0,08–5,37)	
Tile	16 (5,86)	1 (5,88)	15 (5,86)	1,01 (0,10–7,42)	
**Wall material**					
Wood	245 (89,74)	16 (94,12)	229 (89,45)	1,92 (0,22–12,85)	
Brick wall	81 (29,67)	4 (23,53)	77 (30,08)	0,71 (0,20–2,35)	
**Household location**					
Rural	148 (54,21)	8 (47,06)	140 (54,69)	0,70 (0,22–2,02)	
Urban	125 (45,79)	9 (52,94)	116 (45,31)	1,00	
**Household proximity**					
Very near	64 (23,44)	6 (35,29)	58 (22,66)	1,00	
Near	80 (29,30)	5 (29,41)	75 (29,30)	0,62 (0,15–2,25)	
Scattered	74 (27,11)	3 (17,65)	71 (27,73)	0,38 (0,08–1,73)	
Very scattered	55 (20,15)	3 (17,65)	52 (20,31)	0,48 (0,09–2,30)	
**Characteristics of peridomiciliary area**					
**Vegetation**					
Bushes	255(93,41)	15(88,24)	240(93,75)	0,61 (0,10–3,23)	
Trees	254(93,04)	15(88,24)	239(93,36)	0,51 (0,09–2,49)	
Grasses	147(53,85)	7(41,18)	140(54,69)	0,59 (0,20–1,69)	
**Crops**					
Yucca	21(7,69)	1(5,88)	20(7,81)	0,76 (0,08–5,80)	
Corn	4(1,47)	0(0)	4(1,56)	NE	
Cacao	12(4,40)	1(5,88)	11(4,30)	1,40 (0,14–9,66)	
Tomato	5(1,83)	0(0)	5(1,95)	NE	
Name	9(3,30)	0(0)	9(3,52)	NE	
**Animals**					
Poultry	200 (73,26)	9 (52,94)	191 (74,61)	0,37 (0,12–1,09)	
Porcine	134 (49,08)	6 (35,29)	128 (50,00)	0,50 (0,16–1,54)	
Donkey	95 (34,80)	6 (35,26)	89 (34,77)	0,96 (3,07–2,85)	
Opossum	151(55,51)	8(47,06)	143(56,08)	0,96 (0,23–1,91)	
Turkey	71 (26,01)	4 (23,53)	67 (26,17)	0,81 (0,22–2,80)	
Horse	80 (29,30)	6 (35,29)	74 (28,91)	1,24 (0,40–3,61)	
Mule	31 (11,36)	2 (11,76)	29 (93,55)	1,03 (0,19–4,85)	
**Presence of animals in intra-domiciliary area**					
Canines	157 (57,51)	8 (47,06)	149 (58,20)	0,62 (0,21–1,76)	
Felines	169 (61,90)	13 (76,47)	156 (60,94)	0,46 (0,62–6,63)	
Rats	212 (77,66)	14(82,35)	198(77,34)	1,36 (0,34–4,89)	
**Tick infestation**					
**Practices to prevent tick infestation**					
Use of white clothes for outdoor working	76 (27,84)	5(29,41)	71(27,73)	1,10 (0,34–3,35)	
Use of long sleeved shirts in outdoor working	207 (75,82)	12(70,59)	195(76,17)	0,27 (0,22–2,25)	
Tick removal after outdoors working	157 (57,51)	5 (29,41)	152 (59,38)	0,27 (0,08–0,88)	0.26 (0.08–0.84)
Use of insect repellents	37 (13,55)	2 (11,76)	35 (13,67)	0,84 (0,19–3,21)	
Forest fragmentation and deforestation	171 (62,64)	11 (64,71)	160 (62,50)	1,01 (0,35–3,12)	

The variables fever in the last year and removal of ticks after working outdoors were retained in the final model. The model showed that people who removed ticks after working outdoors had a 73.4% lower probability of being incident to rickettsial infection compared to people who do not remove their ticks (RR = 0.27, 95%CI 0.08–0.84). In addition, people reporting fever episodes in the last year had 4.26 –fold risk of being incident to rickettsial infection compared to people not reporting fever episodes (RR = 4.26, 95%CI: 1.16–9.31) (Table 6).

## Discussion

Cohort studies addressing rickettsioses or rickettsial infection are not being conducted in Colombia because of the difficulty in the follow-up of participants and their costs. This study represents an approximation to the incidence of SFG rickettsial infection in a 12-month period and its associated factors in a rural area of Colombia.

Historically, rickettsioses outbreaks in Colombia occur in several regions, are sporadic, and have a high lethality [1,2,19]. These characteristics make it challenging to obtain records of new cases of the diseases, and thus to estimate the incidence of the disease. In Colombia, rickettsioses is a neglected disease, it is not included in the surveillance system and consequently most of the sporadic cases are deadly. In the present study, the estimation of the cumulative incidence of rickettsial infection in the Urabá region demonstrated circulation of rickettsiae in this area, suggesting that these agents might be related to the febrile syndromes reported in the region.

Similar findings were reported in North Carolina, United States, where a prospective study to estimate the infection risk by rickettsial agents in outdoor workers reported new cases of infections caused by *Anaplasma*, *Erlichia*, and *Rickettsii*. In that study, it was possible to identify the probable agents infecting the workers and estimate their incidence: 6% incidence for *R*. *rickettsii*, 12% for *R*. *parkeri* and 9% for *R*. *amblyommatis* [20].

However, it was not possible to identify the rickettsiae species probably infecting humans in the present study, perhaps because of the differences in the immune response of people or frequent exposures to diverse rickettsial agents, which did not allow detecting a four-fold increase in antibody end point titers against rickettsiae antigens. Additional assays would be useful to improve the diagnosis of rickettsial species involved, such as Western Blot or cross absorption test [21].

The National Notifiable Diseases Surveillance System in the United States reported an annual incidence of six cases of rickettsioses (Rocky Mountain spotted fever) per million inhabitants [22–24]. Although a low incidence of the disease was estimated, it is necessary to underscore the high lethality of *R*. *rickettsii*, and thus the importance of early diagnosis and treatment in endemic areas where rickettsioses have become a priority [4].

Remarkably, in the United States a decrease in the incidence of severe cases of rickettsioses has been reported, related to the geographical expansion of *Amblyomma americanum*, which is the vector of *R*. *amblyommatis* [25]. Similarly, the detection of *R*. *amblyommatis* in ticks in the Urabá region suggest that this species might cause mild cases of rickettsioses and thus be protective against severe infections with *R*. *rickettsii*.

Of note, detection of *R*. *rickettsii* and *R*. *amblyommatis* in equines, which are considered as sentinels of rickettsial infection in areas of Brasil and Colombia [10,13], demonstrated the circulation of these rickettsial species in the Urabá region. Consequently, an underdiagnoses of cases of rickettsioses is possible considering that the disease is not notifiable to the surveillance system in Colombia, and it is not included among the differential diagnosis of febrile syndromes. Most cases associated with rickettsial species other than *R*. *rickettsii* manifest with regional adenopathies, rash and eschar at the tick bite-site, such as in *R*. *parkeri* infection [26]. In the present study, incident cases of rickettsial infection most likely were caused by rickettsiae of low pathogenicity and presented a mild clinical syndrome with myalgia, adenopathy, and skin lesions. However, diagnosis of these rickettsiae of low or unknown pathogenicity, such as *R*. *amblyommatis* [21,25], was not achieved because participants refused the collection of a second blood sample in the follow-up year or because limitations of study personnel.

Results showed that people checking their selves after working outdoors had a lower risk of infection with rickettsiae. This is in agreement with CDC recommendations to prevent rickettsial infection, such as the use of white clothes, long sleeved shirt and long pants, insect repellents, and checking their body to remove ticks attached to the skin or clothes for people living or spending time in areas at risk of rickettsial transmission, such as forests, mainly between May and August [4]. In the study area, it is not clear if people used long sleeved shirt and long pants for sun protecting or as prevention measure to avoid tick-bites.

It is important to highlight that in some areas where equines were infected with rickettsiae, infected people who live or work in the same area were also detected. For instance, in Caracolí hamlet in Alto de Mulatos, one person who was incident for rickettsial infection owned two equines, one *E*. *caballus* and other *E*.*asinus x E*. *caballus* that were also incident for rickettsial infection. Likewise, several equines incident for rickettsial infection (*R*. *rickettsii* and *R*. *amblyommatis*) that were detected in the urban center of Alto de Mulatos and the hamlet La Unión from Las Changas, were located near households of incident people from the urban center of Alto de Mulatos and hamlet La Salada (close to hamlet La Unión). These results support the circulation of rickettsial species in rural areas of Urabá region.

Moreover, it is necessary to conduct additional qualitative studies in the area to understand more in-depth the social cultural processes related to rickettsioses. The knowledge about rickettsiae circulation in different hosts and potential vectors is a starting point from where to implement education programs in communities that have been affected by rickettsiosis. Health education is of crucial importance to prevent the more serious cases of the disease.

The main limitation of the present study was the loss to follow-up in the second sampling in both areas of Alto de Mulatos and Las Changas. The loss to follow-up was the result of people moving to other regions in search of work after the harvesting season in the area. In addition, people were reluctant to provide the second blood sample, because they thought it could be debilitating or people were not interested in participating after a negative result in the first sampling period. These selection bias could affect the identification of additional factors related to rickettsial transmission such as outdoors working, materials of households associated to tick infestation and the presence of wild animals in peri-domiciliary area, among others. Further, the selection bias possibly underestimated the incidence of infection because most of the people lost to the follow up at 12 months were outdoors workers and consequently they were more exposed to tick infestation than people working indoors. Another limitation was the poor knowledge about the specificity and sensitivity of the immune-fluorescent test to detect asymptomatic cases of rickettsial infection. This could likely affect the detection of rickettsial incident cases in both areas. Finally, information bias could be present in participants of the second sampling, such as memory bias, when providing information about fever and tick infestation in the year of follow-up.

### Conclusions

The results showed a recent circulation of SFG rickettsiae in the study area where previous lethal outbreaks of the disease were reported. However, the cumulative incidence of rickettsial infection in people was considered low. In addition, both people and equines from the study area were exposed to rickettsiae infections. The species infecting equines were probably *R*. *rickettsii*, *R*. *amblyommatis* or *R*. *belli*, which underscores the role of equines as sentinels of infection.

The estimated association between the incidence of rickettsial infection and fever episodes suggests that most rickettsioses cases are underdiagnosed, and consequently are included as unspecified fever in hospital surveillance records. In addition, people with protective habits against tick infestation (removal of ticks after working outdoors) had a lower risk of infection with SFG rickettsiae in these rural areas.

Finally, the results of this study are the first approach for the design and implementation of surveillance programs of rickettsioses from the spotted fever group in areas where previous lethal outbreaks were reported. These programs can benefit from the collaboration between government entities and research groups working in rickettsioses in Colombia.

## Supporting information

S1 Checklist Strobe checklist for cohort studies. (DOCX)
